# Breed predisposition to canine gastric carcinoma - a study based on the Norwegian canine cancer register

**DOI:** 10.1186/1751-0147-55-25

**Published:** 2013-03-21

**Authors:** Tonje Seim-Wikse, Einar Jörundsson, Ane Nødtvedt, Tom Grotmol, Charlotte R Bjornvad, Annemarie T Kristensen, Ellen Skancke

**Affiliations:** 1 Department of Veterinary Clinical and Animal Sciences, Faculty of Health and Medical Sciences, University of Copenhagen, Frederiksberg, Copenhagen, Denmark; 2 Department of Basic Sciences & Aquatic Medicine, Norwegian School of Veterinary Science, Oslo, Norway; 3 Department of Companion Animal Clinical Sciences, Norwegian School of Veterinary Science, Oslo, Norway; 4 Cancer Registry of Norway, Oslo, Norway

**Keywords:** Dog, Gastric carcinoma, Breed, Proportional morbidity ratio, Cancer registration

## Abstract

**Background:**

Previous research has indicated a breed predisposition to gastric carcinoma in dogs. However, results to date are inconsistent since several studies have failed to prove such a predisposition. Better knowledge of breeds at risk could facilitate early detection of gastric carcinoma in dogs. The aim of the study was to retrospectively investigate the proportion and possible breed predisposition to canine gastric carcinoma using the Norwegian Canine Cancer Register for calculations of proportional morbidity ratios (PMRs) for the period 1998–2009.

**Results:**

Histologically verified tumours recorded in the Norwegian Canine Cancer Register were studied (n = 19,715). A total of 31 (0.16%) cases of canine gastric carcinomas were identified. The median age of affected dogs was 10 years. The most commonly reported clinical signs were vomiting, anorexia, and weight loss. Males had significantly higher odds of gastric carcinoma than females (P = 0.02). The PMR with 95% confidence interval (CI) was calculated for each breed, and a breed predisposition was identified. Individuals of the breeds Tervuren (PMR 56.1), Bouvier des Flandres (PMR 36.5), Groenendael (PMR 34.5), Collie (PMR 26.1), Standard poodle (PMR 7.6), and Norwegian elkhound (PMR 6.1) had a significantly increased risk of developing gastric carcinoma.

**Discussion and conclusion:**

The proportion of cases of gastric carcinoma recorded in the Norwegian Canine Cancer Register was found to be 0.16%, and a breed predisposition was identified. The breed predisposition observed in the current study indicates a genetic susceptibility to gastric carcinoma.

## Background

Canine gastric cancer is rarely diagnosed and is reported to account for approximately 0.1–0.5% of canine neoplasias [1-3]. However, it is still more prevalent in dogs than in other domestic animals [4]. The majority of gastric malignancies in dogs are carcinomas, accounting for 50–90% [2,5,6], followed by leiomyosarcomas and malignant lymphoma [2]. Adenocarcinoma is the most common type of gastric carcinoma [2]. This subtype of carcinoma forms tubular structures and may exhibit various patterns at different levels of the invasion of the stomach wall. Undifferentiated carcinomas, which comprise the remaining carcinomas, have no visible glandular structures [4]. The clinical relevance of these histopathological subtypes of carcinoma is unknown, and the general term ‘gastric carcinoma’ is used in this article. Among dogs, gastric carcinoma has been reported to affect males more frequently than females [2,3,6-9]. A higher incidence among males is also seen for human gastric carcinoma [10]. Gastric carcinoma is generally a disease of older dogs, but has been reported in dogs in the age range 3–20 years, with a mean age at diagnosis of 8.4–10.8 years [2,3,5,7-9,11,12]. The incidence of gastric carcinoma increases with age also in humans [10]. Gastric carcinoma in dogs is most often located in the lesser curvature and the pyloric region of the stomach [2,3,5,8].

The most common clinical signs associated with gastric carcinoma are vomiting, anorexia, and weight loss. Haematemesis, melaena, anaemia, lethargy, ptyalism, polydipsia, abdominal distension, and abdominal discomfort are other reported signs [3,5,6,8,9]. The clinical signs resemble those associated with chronic gastritis [13]. The majority of canine patients have had clinical signs for approximately four months or less by the time of diagnosis, although as much as 18 months’ duration is reported [3,5,6,8,14]. The prognosis in cases of gastric carcinoma is poor. One case series reported a median survival time of 35 days, with a range of 0 days to 10 months after diagnosis [5]. Furthermore, 70–90% of gastric carcinomas have metastasized by the time of diagnosis or euthanasia [3,5,7]. The most common sites reported for metastases are the regional lymph nodes, with others being the omentum, duodenum, liver, pancreas, spleen, oesophagus, adrenal glands, and lungs [3,5,7,9].

A preliminary diagnosis of canine gastric carcinoma is usually obtained by ultrasound or endoscopic examination of the stomach. Using ultrasound, thickening of the gastric wall, loss of gastric wall layering, and enlargement of regional lymph nodes is likely to be observed [15]. Endoscopy allows visualization of the mucosa and performing biopsies in order to enable a definite diagnosis. A distinct ulcer with thick and irregular walls elevated from the surroundings is commonly seen, but more diffuse changes with loss of rugal folds and submucosal vascular pattern in the absence of ulcers are also described [3,8].

In humans, 95% of gastric cancers are sporadic cases, and *Helicobacter pylori* infection is the most important environmental risk factor [16]. Familial aggregation in a small proportion of cases suggests a significant genetic predisposition [17,18]. Hereditary diffuse gastric cancer in humans, where the causative germ line mutation has been identified as *CDH1*, is reported to be associated with 5% of all gastric cancer [17].

Canine breed predisposition has been reported for some types of cancer [1]. Although breed predisposition to gastric carcinoma has been suspected, several studies have failed to provide supporting evidence [2,5,9]. Table 1 provides a summary of predisposed breeds reported from other studies, the majority of which are case series. These case series are purely descriptive studies, with no comparison between the characteristics of subgroups. Due to this limitation, no assumption about associations between exposure and outcome can be made [19]. Other researchers have performed case control studies based on hospital records [3,12]. No knowledge is available regarding the population-at-risk from which the cases arise in this design. Hence, they cannot be used for estimations of cancer incidence, although the odds ratio of cancer by breed can be estimated [20].

**Table 1:** Previous studies’ results related to breed predisposition to gastric carcinoma in dogs

**Authors**	**Cases**	**Country**	**Breed predisposition**	**Study design**	**Comparison**
1. (Lingeman et al. [9])	61	USA	**No breed** predisposition	Case series	
2. (Sautter et al. [6])	14	USA	**No breed** predisposition	Case series	
3. (Patnaik et al. [2])	26	USA	**No breed** predisposition	Case series	
4. (Sullivan et al. [3])	31	United Kingdom	7/31 **Rough collie** and 3/31 **Staffordshire bull terrier**	Case control	Comparison with breeds of the hospital accessions
5. (Fonda et al. [8])	15	Italy	Suggested genetic mechanism in 8 Belgian shepherd dogs **(7 Groenendael and 1 Tervuren)**	Case series	Pedigree examined in 8 cases
6. (Scanziani et al. [7])	11	Italy	High incidence in Belgian shepherd dogs **(Groenendael)**	Case series	
7. (Penninck et al. [12])	16	USA	4/16 cases **chow-chow** (RR 46.2)	Case control	Relative prevalence determined against hospital population
8. (Swann and Holt. [5])	19	USA	**No breed** predisposition	Case series	
9. (Bilek and Hirt. [11])	19	Austria	3/19 **chow-chow** (OR 23.5)	Case control	Comparison with numbers from the Austrian Kennel Club
10. (Qvigstad et al. [21])	4	Norway	**Norwegian Lundehund**	Case series	
11. (Lubbes et al. [10])	92	The Netherlands	Incidence: 1,18% among **Tervuren**	Retrospective cohort	

Due to the advanced stage of disease at time of diagnosis and the high frequency of metastasis, early detection is essential if treatment of canine gastric cancer is to be attempted. Because presenting signs may be similar to those observed in cases of chronic gastritis, some patients are symptomatically treated for gastritis for prolonged periods. The suspicion of gastric cancer may first arise when this form of treatment fails. It is therefore important for animal welfare to obtain a correct diagnosis as early as possible. Better knowledge of breeds at risk can enable early detection of gastric carcinoma in affected cases and may also facilitate comparative genetic studies that could identify factors contributing to the development of gastric cancer.

We hypothesized that breed predisposition to canine gastric carcinoma exists in dogs. The aim of the study was to retrospectively investigate the proportion and possible breed predisposition to canine gastric carcinoma using data from the Norwegian Canine Cancer Register as a basis for calculating PMR for the period 1998–2009.

## Materials and methods

### Study population

Data were retrieved from the Norwegian Canine Cancer Register, a database consisting of all histopathologically verified tumours submitted to the diagnostic pathology laboratory at the Norwegian School of Veterinary Science between March 1998 and December 2009. The samples originated from private veterinary clinics all over Norway as well as from the Norwegian School of Veterinary Science. Information regarding each dog’s name, breed, sex, age in years, and histological diagnosis is included in the database. The original referral forms were searched for information on methods of sampling, clinical signs, duration of signs, and nutritional status. The Norwegian Lundehund has been reported to be predisposed to gastric carcinoma [21], but gastrointestinal samples from Norwegian Lundehunds were not included in the registry for this time period due to a separate project involving this breed. The database was searched for the terms ‘neoplasia’ and ‘stomach’, and the combination of the two terms was defined as ‘gastric cancer’.

### Histopathological examination

Histopathological examination was performed on formalin-fixed, paraffin-embedded tissue specimens stained with haematoxylin-eosin and van Gieson’s. All histological slides classified as gastric cancer in the database were re-examined by an experienced veterinary pathologist (EJ) during 2011. Classification was performed according to the World Health Organization standard [4]. The pathologist was blind to the original histopathological diagnosis when reviewing the slides. Immunohistochemistry with cytokeratin and vimentin was performed if there was uncertainty regarding the diagnosis. The specimens diagnosed as carcinomas were included as cases in the study.

### Statistical analysis

The number of gastric carcinomas in the registry was divided by the number of all other submitted tumours in order to yield the overall proportion of gastric carcinomas. The PMR of gastric carcinoma by breed was calculated by dividing the number of gastric carcinomas in a breed (*a*_1_) by all tumours in the breed (*n*_1_), over the number of gastric carcinomas in all other breeds (*a*_0_) divided by all other tumours in the other breeds in the database (*n*_0_) = (*a*_1_/*n*_1_)/(*a*_0_/*n*_0_) [22]. The 95% CI for the PMR was computed on a logarithmic scale [23]. The odds ratio (OR) for gastric carcinoma was calculated for males compared to females, and the P-value was estimated based on Fisher’s exact test. The level of statistical significance was set at P < 0.05. All statistical analyses were performed using the software package Stata, Version 11 (Stata Corporation, College Station, Texas, USA).

## Results

The material consisted of 19,715 tumours, of which 44 had been diagnosed as gastric cancer. In total, 10/44 (22.7%) were given a new histopathological diagnosis when re-examined during 2011. 31/44 were confirmed as gastric carcinomas (0.16% of total). The 13 cases which had been diagnosed with gastric cancer in the register, and which were not diagnosed as carcinomas, were found to be two carcinoma in situ, one adenoma, one hyperplasia, two metastasized tumours with unknown primary tumours, and six round cell tumours. In one case the diagnosis was inconclusive due to small and inadequate biopsies. Adenocarcinoma was the most prevalent carcinoma, occurring in 23/31 (74%) of the cases, whereas 8/31 (26%) were undifferentiated carcinomas. In total, 14 of the 31 cases originated from the Norwegian School of Veterinary Science, and the remaining 17 cases originated from private clinics.

The sex of the affected dog was known for 19,489 tumours; the sex distribution is presented in Table 2. The OR for males versus females was 2.3 (P < 0.02). The male to female ratio was 1.2:1. The age at diagnosis of a gastric tumour ranged from 1 year to 13 years, with a median age of 10 years. In 17 out of 31 cases the tumour had been sampled by gastroscopy. The remaining biopsies had been performed during exploratory laparotomies or during post-mortems. The gastric location of the tumour was described for 19 of the cases and was restricted to the lesser curvature and/or pyloric region in 18 cases (95%). In one case (5%), the tumour was observed in the cardiac region.

**Table 2:** Sex distribution among dogs with gastric carcinoma, recorded in the Norwegian canine cancer register (1998–2009)

	**Cases**	**Non-cases**	**Total**
Male	17 (0.25%)	6,680 (99.75%)	6,697
Female	14 (0.11%)	12,778 (99.89%)	12,792
	31 (0.16%)	19,456 (99.83%)	19 489

Based on the referral sheets from the submitting veterinarian, clinical information was obtained for a subset of the included cases. The nutritional status was described in 16 cases, and characterized as either ‘obese’ (1/16; 6%), ‘good/normal’ (5/16; 31%), or ‘poor/thin’ (10/16; 63%). In 16 cases, the clinical signs were described. All of the dogs had experienced vomiting (16/16; 100%), and haematemesis had occurred in only 2/16 (13%) cases. Anorexia was observed in 6/16 (38%) cases and weight loss in 5/16 (31%) cases. Other, less frequently described signs were drooling, lethargy, diarrhoea, reduced general condition, abdominal pain, and polydipsia. The clinical signs are summarized in Table 3. The duration of signs prior to biopsy or euthanasia was usually described in terms of ‘weeks’ or ‘months’, but in 15 cases the duration was specified and ranged from 2 weeks to 6 months, with a median of 2 months.

**Table 3:** Clinical signs in dogs with gastric carcinomas recorded in the Norwegian canine cancer register (1998–2009) (n = 16)

Vomiting	16
Anorexia	6
Weight loss	5
Haematemesis	2
Drooling	2
Lethargy	2
Reduced general condition	2
Melena	1
Abdominal pain	1
Polydipsia	1

Table 4 shows the total number of gastric carcinomas and PMR with 95% CI by breed. All samples from Tervuren (n = 7) and Groenendael (n = 3) breeds were diagnosed as adenocarcinomas. The value 1 represents the ratio for the average breed. Breeds in which the 95% CI did not include 1 were considered to have significantly increased (or decreased) risk of gastric carcinoma. The breeds with significantly increased risk were found to be Tervuren, Bouvier des Flandres, Groenendael, collie (rough or smooth not specified), standard poodle, and Norwegian elkhound.

**Table 4:** Cases of gastric carcinoma, tumours, and PMR by breed, recorded in the Norwegian canine cancer register (1998–2009) (n = 31)

**Breed**	**Gastric carcinomas**	**All tumours in the breed**	**PMR**	**95% CI low**	**95% CI high**
Tervuren	7	102	56.1	24.7	127.2
Bouvier des Flandres	1	18	36.5	5.3	253.3
Groenendael	3	61	34.5	10.8	110.5
Collie	2	52	26.1	6.4	106.5
Standard poodle	3	273	7.6	2.3	25.0
Norwegian elkhound	2	219	6.1	1.5	25.6
Nova Scotia duck tolling retriever	1	118	5.5	0.8	40.3
Leonberger	1	124	5.3	0.7	38.3
Border collie	1	331	2.0	0.3	14.3
Bichon fries	2	434	3.1	0.7	12.8
Dachshund	2	534	2.5	0.6	10.4
Gordon setter	2	544	2.4	0.6	10.2
Irish setter	1	349	1.9	0.3	13.5
Labrador retriever	1	616	1.0	0.1	7.6
German shepherd	1	1194	0.5	0.1	3.8
English setter	1	1407	0.4	0.1	3.2

## Discussion

In the present study, 0.16% of all canine tumours submitted to the diagnostic pathology laboratory at the Norwegian School of Veterinary Science during the period March 1998 to December 2009 were gastric carcinomas. This proportion corresponds to what Arnesen et al. (2001) found in their study of the Norwegian Canine Cancer Register from 1990–1998 [1].

Breed predisposition to gastric carcinoma was detected based on the PMR. The highest relative proportion of gastric carcinoma was observed in Tervuren, Bouvier des Flandres, Groenendael, collie (rough or smooth not specified), standard poodle, and Norwegian elkhound. Tervuren had a PMR of 56, indicating that this breed is 56 times more likely to be diagnosed with gastric carcinoma than the average breed in this database. Belgian shepherd dogs are bred as four separate types – Tervuren, Groenendal, Mallanois, and Lakenois – but breeding between these types is permitted by the Norwegian Belgian shepherd dog club (NBFK) when specific conditions are fulfilled and with limited frequency. Registration of offspring is determined by the length, colour, and structure of the coat [24]. Even though the four types are closely related, there are no reports of increased risk of gastric carcinoma in the Mallanois or the Lakenois. It is unknown whether this is due to the Mallanois and Lakenois being less frequent types of Belgian shepherd dog or whether there is a different prevalence between the types.

Belgian shepherd dogs, both Groenendael and Tervuren types, and rough collies have previously been reported to be at increased risk of developing gastric carcinoma [3,7,8,25]. Single cases of gastric carcinoma have previously been reported in Norwegian elkhound, standard poodle, and Bouvier des Flandres [5,6,12], but the present study is the first time that these breeds have been found to be at risk of this type of cancer. In this study, the breed Bouvier des Flandres was found to have an increased risk of developing gastric carcinoma, but the wide 95% CI for PMR renders the result uncertain, and it may not be clinically significant.

It is worth noting that the breeds boxer, Rottweiler, and flat-coated retriever were not among the cases of gastric carcinoma in our study. These are all common breeds in Norway, and in previous studies based on the Norwegian Canine Cancer Register they were reported to have the highest relative risk ratio (RR) for developing tumours in general [1]. The database utilized for the current study included 616 tumour cases in boxers, 514 in Rottweilers, and 1,052 in flat-coated retrievers. A further interesting observation is the lack of mixed breeds among the cases of gastric carcinoma, although there are 1,932 tumours from mixed breeds in the database, making ‘mixed’ the most frequent ‘breed’. Despite being the most common pure-bred dog in the database, with 1,407 tumours, the English setter is only represented with a single case. Chow chow and Staffordshire bull terrier breeds have previously been reported to have an increased risk of gastric carcinoma [3,11]. Both breeds were found to be infrequent in the database, with 14 tumour cases in chow chows and 22 tumour cases in Staffordshire bull terriers during the period 1998–2009. Neither of these breeds are represented in the current study material. This may be because these breeds are relatively uncommon in Norway, or that they have a different genetic composition compared to dogs from the same breeds in other countries.

Males were found to have significantly higher odds of gastric carcinoma than females in our study. This finding is in agreement with results from other studies and similar to what has been described in humans [2,3,6-10].

All dogs with gastric carcinoma in the current study, where clinical information was available, experienced vomiting. This, together with weight loss and anorexia, were the most commonly reported clinical signs. The complete clinical symptomatology may have been underreported, as a complete veterinary medical record was not available for all cases. In several cases, ‘vomiting’ was the only clinical sign reported, as this probably has been the main reason for diagnostic work-up, even though other clinical signs may have been present. The described clinical signs are in accordance with those reported in earlier studies [3,5,6,8,9].

Dog breeds arise from a limited number of ancestors and combined with a frequent use of popular males, each domestic breed is an isolated population [26,27]. The genetic diversity within breeds is reduced, but there is a greater genetic divergence between breeds [26]. This makes the performance of genetic studies of dogs easier than those conducted in complex populations. Dogs share environments with their owners. Furthermore, because they naturally develop spontaneous cancers, in contrast to mouse models, they constitute an excellent comparative model for cancer [27]. The results from the study, identifying several breeds at risk, suggest that a genetic predisposition is of greater significance in dogs than humans, who have experienced a rapidly declining incidence of gastric carcinoma due to recent changes in the environment [16]. The dog may thus be a useful model for further comparative genetic studies of gastric cancer.

If differences in the ability of veterinarians to diagnose a gastric tumour and the willingness of dog owners to pursue and pay for diagnostic work-up are related to dog breed, they may influence the results of our study and be a source of selection bias. Underestimation can occur as a result of false negative biopsies. There might also be an underestimation of the prevalence of gastric cancer, as advanced disease and the age of an affected dog can result in owners being reluctant to pursue further diagnostic examinations.

A strength of the present study is the use of PMR to assess the proportion of all the affected animals in the population that have a particular disease under question. This allowed comparison with the background population of submitted cases, as opposed to the case series commonly reported in veterinary literature. However, a weakness of PMR is its sensitivity to variations in breed popularity and uncertainty in the estimates related to uncommon breeds. This is further influenced by the low prevalence of the disease. This might explain why some breeds previously described to have a higher risk are not represented in this study. It is also possible that a larger study would have been able to detect more breeds at increased risk among Norwegian dogs.

In the Norwegian Canine Cancer Register there is a higher proportion of females than males. This is probably due to mammary tumours dominating the register, as reported in previous studies [1]. Of the 44 cases that were diagnosed with gastric cancer in the Norwegian Canine Cancer Register, 22.7% were given a new histopathological diagnosis when they were re-evaluated in 2011. The proportion of cases given a new histopathological diagnosis corresponds to that found in previous studies based on the Norwegian Canine Cancer Register [28].

The breed predisposition observed in the current study indicates a genetic susceptibility to gastric carcinoma. This is in contrast to gastric cancer in humans, which in developed countries has declined significantly in incidence over the past 50 years, attributed largely to the eradication of *H pylori* infection [16]. A reasonable interpretation is that environmental risk factors play a smaller role in the development of gastric cancer in dogs compared to humans.

## Conclusion

The proportion of cases of gastric carcinoma recorded in the Norwegian Canine Cancer Register for the period March 1998 to December 2009 was found to be 0.16%. A breed predisposition to gastric cancer was identified in the study, namely Tervuren, Bouvier des Flandres, Groenendael, collie, standard poodle, and Norwegian elkhound were found to have a significantly increased risk of gastric carcinoma according to the proportional morbidity ratios (PMRs).

## Abbreviations

PMR: Proportional morbidity ratio; OR: Odds ratio; CI: Confidence interval.

## Competing interest

All authors declare that they have no competing interests.

## Authors’ contribution

TSW participated in the design of the study, preparing data from the database and performed the statistical analysis and drafted the manuscript. EJ took part in designing the study and evaluated all the histological slides. AN took part in designing the study, helped with the statistical analysis and revised the manuscript. CB, TG and AMK participated in designing the study and revision of the manuscript. ES took part in designing the study, collected parts of the data and revised the manuscript. All authors read and approved the final version of the manuscript.
